# An Identification Method for Road Hypnosis Based on XGBoost-HMM

**DOI:** 10.3390/s25061842

**Published:** 2025-03-16

**Authors:** Longfei Chen, Chenyang Jiao, Bin Wang, Xiaoyuan Wang, Jingheng Wang, Han Zhang, Junyan Han, Cheng Shen, Kai Feng, Quanzheng Wang, Yi Liu

**Affiliations:** 1 College of Electromechanical Engineering, Qingdao University of Science and Technology, Qingdao 266000, China; chenlongfei@mails.qust.edu.cn (L.C.); jiaochenyang@mails.qust.edu.cn (C.J.); wangbin@mails.qust.edu.cn (B.W.); zhanghan@mails.qust.edu.cn (H.Z.); hanjunyan@mails.qust.edu.cn (J.H.); b024030003@mails.qust.edu.cn (C.S.); fengkai@mails.qust.edu.cn (K.F.); 0020030005@mails.qust.edu.cn (Q.W.); yiliu@mails.qust.edu.cn (Y.L.); 2 Department of Mathematics, Ohio State University, Columbus, OH 43220, USA

**Keywords:** road hypnosis, driver, HMM, XGBoost, state identification, vehicle

## Abstract

Human factors are the most important factor in road traffic crashes. Human-caused traffic crashes can be reduced through the active safety system of vehicles. Road hypnosis is an unconscious driving state caused by the combination of external environmental factors and the driver’s psychological state. When drivers fall into a state of road hypnosis, they cannot clearly perceive the surrounding environment and make various reactions in time to complete the driving task, and driving safety is greatly affected. Therefore, road hypnosis identification is of great significance for the active safety of vehicles. A road hypnosis identification model based on XGBoost—Hidden Markov is proposed in this study. Driver data and vehicle data related to road hypnosis are collected through the design and conduct of vehicle driving experiments. Driver data, including eye movement data and EEG data, are collected with eye movement sensors and EEG sensors. A mobile phone with AutoNavi navigation is used as an on-board sensor to collect vehicle speed, acceleration, and other information. Power spectrum density analysis, the sliding window method, and the point-by-point calculation method are used to extract the dynamic characteristics of road hypnosis, respectively. Through normalization and standardization, the key features of the three types of data are integrated into unified feature vectors. Based on XGBoost and the Hidden Markov algorithm, a road hypnotic identification model is constructed. The model is verified and evaluated through visual analysis. The results show that the road hypnosis state can be effectively identified by the model. The extraction of road hypnosis-related features is realized in non-fixed driving routes in this study. A new research idea for road hypnosis and a technical scheme reference for the development of intelligent driving assistance systems are provided, and the life identification ability of the vehicle intelligent cockpit is also improved. It is of great significance for the active safety of vehicles.

## 1. Introduction

About 1.2 million people die in road traffic crashes every year, and traffic crashes are the second most common cause of death [1,2]. The number of traffic deaths has increased by 5% per year over the past decade, while the population growth rate is only 1.4% [3]. It is shown that a significant correlation exists between traffic crashes and driver factors [4]. According to the analysis, 78% of traffic crashes are caused by driver factors [5]. Among them, 25% of traffic crashes are caused by distracted driving, and 20% of traffic crashes are caused by fatigue driving [6,7]. In addition, emotions [8,9,10,11], drunk driving [12,13], drug driving [14], and elderly driving [15] are also important factors in traffic crashes.

In 1963, Williams found that when the correct driving posture was maintained in drivers and they drove in a monotonous road environment, a state similar to hypnosis was entered [16]. Williams believes that this hypnosis-like state is manifested by the driver’s gaze on the road line or a fixed point ahead. As a result, drivers cannot recognize the dangerous situation during the driving process and take timely measures [17]. Brown further explained that even if the correct sitting posture was maintained, the eyes were focused forward, and the hands were placed on the steering wheel, a hypnosis-like phenomenon would still be experienced by the driver [18]. Subrahmanyan called this phenomenon “White Line Fever” and described it as a mild hypnotic or tranced state. It usually occurs when driving long distances, especially on monotonous straight roads [19]. Although drivers may be physically awake, their consciousness may not be fully focused. Kerr further described this phenomenon as a state of “Driving Without Awareness mode” [20]. Driving Without Awareness (DWA) refers to the driver’s conscious state close to hypnosis when he feels tired. Even though his eyes are open, he cannot notice the surrounding traffic. At this time, the driver may experience a “trance” state [21]. Through analysis of road geometry, Khotimah found that drivers are more likely to feel fatigued and sleepy on straight and monotonous roads [22]. Thiruvalara’s study found that drivers might enter a hypnosis-like state when staring at the road for a long time. Although normal driving can be maintained, there is no memory of the journey [23]. Cerezuela found that long periods of driving on highways and regular roads could lead to different predictability in visual stimulus motion patterns. Their alertness may be reduced, especially in the later stage of driving [24]. Through virtual driving experiments, Briest found that some drivers might enter a deep unconscious driving state in monotonous environments like highways. This is an obvious state of unconsciousness. At this time, the DWA mode will also appear [25]. Xiaoyuan Wang et al. conducted in-depth research on this phenomenon and defined it as a road hypnosis phenomenon [26]. Specifically, it is an unconscious driving state formed by the combined effect of external environmental factors and the driver’s psychological state. In this state, although the driver seems to be able to maintain a normal driving state, the reaction speed is obviously slower than the normal driving state. On the basis of clarifying the definition of road hypnosis, Xiaoyuan Wang et al. designed and conducted reasonable vehicle and virtual driving experiments to induce the road hypnosis state in drivers and collect relevant data. The road hypnosis state was preliminarily explored through eye movement features, ECG features, and EMG features [27,28]. Further exploration of the essential characteristics of road hypnosis was conducted with EEG data, a golden indicator for identifying the driver’s life state [29]. A road hypnosis identification model based on the driver’s physiological characteristics was built by integrating EEG data and eye movement data features [30].

The earliest source of this research is the phenomenon of high-speed hypnosis [16,17,18,19,20,21,22,23,24,25]. There are some studies about the existence of highway hypnosis. However, after understanding this phenomenon. With the consideration of the experience in daily driving, we found that in addition to highways, a phenomenon similar to highway hypnosis may also occur in other driving scenarios. Therefore, the concept of “road hypnosis” was proposed and related research was carried out. Our research shows that the road hypnosis phenomenon is not limited to highways. It may occur in any driving scene. Initially, we performed preliminary identification through eye movement recognition on fixed driving routes and scenes. Subsequently, EEG character for identification road hypnosis was gradually introduced. Finally, the experiment scenarios were expanded to the identification of road hypnosis in non-fixed driving routes. Compared with other studies [16,17,18,19,20,21,22,23,24,25], although the phenomenon of high-speed hypnosis has been studied in other studies, in-depth specific identification work has not been carried out in most of them. Compared with our research [26,27,28,29,30], the experiment scene in this research is non-fixed driving routes. Multi-source and diverse parameter factors are also considered in this study. The identification of road hypnosis has been improved.

Based on the above-related research, an identification model for road hypnosis based on non-fixed driving routes is proposed in this study. Vehicle driving experiments on non-fixed driving routes are designed and conducted to collect relevant data on the driver’s road hypnosis state. The collected data are preprocessed, and features are extracted. The preprocessed driver and vehicle data are integrated with local linear embedding (LLE), power spectral density (PSD), and point-by-point calculation methods. A road hypnosis identification model for drivers is constructed based on the XGBoost-HMM algorithm. The model’s performance is evaluated with six indicators, which are mean squared error, coefficient of determination, root mean squared error, mean absolute error, explained variance, and maximum error. The LIME-SHAP algorithm and the K-fold cross-validation method are introduced for the explanation and verification of the model. The experimental results show that the road hypnosis identification model built for non-monotonous road environments effectively identifies the driver’s road hypnosis state.

A new road hypnosis identification method is proposed in this study. The feasibility of extracting and identifying road hypnosis states in multiple driving scenarios is verified. At the same time, the life recognition technology of the smart cockpit is further improved. The active safety system of the vehicle is enriched and improved.

The structure of this study is as follows. Section 1 provides the literature review. The study design and analysis method are presented in Section 2. Section 3 and Section 4 present the results of data processing and discussions, respectively. Finally, concluding remarks are summarized in Section 5.

## 2. Method

### 2.1. Road Hypnosis

Road hypnosis is defined as an unconscious driving state formed by the combined effect of external environmental factors and the driver’s psychological state. It is caused by the repetition and low-frequency stimulation in a highly predictable driving environment. Its specific manifestations are the driver’s perception paralysis, decreased attention, and decreased vigilance, accompanied by temporary trance, amnesia, and fantasy. It is a state that can be induced by multiple factors, such as endogenous factors (the driver’s hypnotic constitution, fatigue, and circadian rhythm) and exogenous factors (road geometry, monotony of driving tasks, monotonous driving situations, and vehicle enclosure). Once the driver is out of the road hypnosis, it is usually accompanied by a clear state of alertness. The driver often does not remember what happened while in the road hypnosis state but has a clear memory of the drowsy state just experienced. Although the driver may appear to maintain normal driving, the reaction speed is significantly slower than in a normal driving state.

Road hypnosis does not require a specific monotonous driving environment. It can occur in any long-duration, highly repetitive driving process, especially when the driver is extremely familiar with the route and it is easy to enter this state. Any road segment that makes the driver feel “there is nothing to do”, regardless of its inherent complexity, may induce a road hypnosis state. The key to the emergence of road hypnosis lies in the driver’s psychological and physiological adaptation, not in the characteristics of the road itself.

### 2.2. Model and Algorithm

In the process of data collection related to road hypnosis in non-monotonous road environments, fewer data are collected for road hypnosis states compared to normal driving states. This causes a category imbalance problem. The transfer mode between potential states in the case of scarce data can be speculated by the Hidden Markov Model (HMM) through its hidden state transfer probability matrix. At the same time, the HMM is well suited for modeling the transitions of hidden states in time-series data, which is suitable for capturing changes in the driver’s latent state. The recognition performance of a few classes can be optimized by the extreme gradient boosting (XGBoost) algorithm through adjusting the sample weight and customizing the loss function, so the performance of the model on the unbalanced dataset can be improved. The XGBoost model performs excellently in accuracy. However, its black box nature as an integrated learning method makes its decision process difficult to interpret. Explanations for individual predictions by local interpretable model-agnostic explanations (LIME) with a local weighted regression model. It reveals the decision-making basis for specific prediction outcomes. The contribution of each feature to the final prediction can be calculated by Shapley additive explanations (SHAP). It offers both global and local-level explanations. This increases the transparency of the relationship between features and predictions.

#### 2.2.1. HMM Algorithm

**Figure 1:**
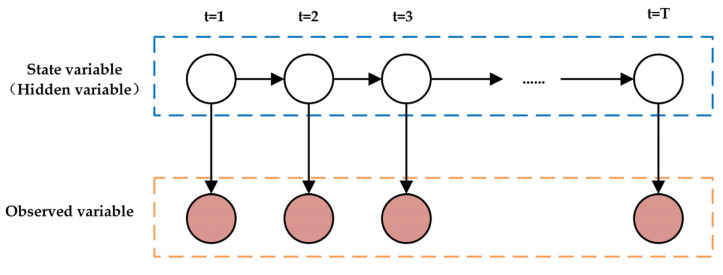
HMM algorithm structure.

The Hidden Markov Model (HMM) is a statistical model used to describe a system consisting of hidden states and observable events [31,32,33]. It is assumed that the states of the system cannot be directly observed. They are inferred through the associated observation data. In addition, the HMM is often used to model the changes in drivers’ hidden states. In the identification of road hypnosis, the HMM can capture the temporal characteristics and latent patterns of driver states. It is suitable for processing data with temporal dependencies. The HMM consists of three main components: hidden states, observation probabilities, and transition probabilities. In the HMM, a transition pattern exists between system states. Each state generates an observation value based on a certain probability distribution. The HMM is widely used in tasks such as time series analysis, speech recognition, and natural language processing. It can model the hidden structure of dynamic systems and infer potential state changes. The change in the driver’s state can be analyzed in road hypnosis detection. The structure is shown in Figure 1.

#### 2.2.2. XGBoost Algorithm

**Figure 2:**
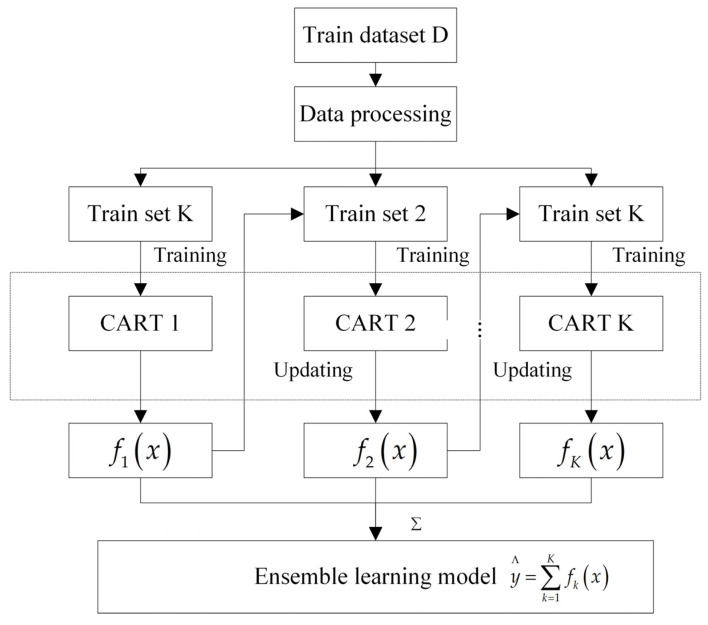
XGBoost algorithm structure.

XGBoost (extreme gradient boosting) is an ensemble learning method based on gradient boosting. It includes ten modules: decision trees, gradient boosting framework, loss function, regularization mechanism, feature splitting, parallel computation, tree pruning, feature importance evaluation, learning rate, and early stopping. Feature importance evaluation can identify the relationship between all input variables and the road hypnosis state. The influence of each feature on the road hypnosis state is determined by examining its contribution to the model [34,35,36,37]. The XGBoost algorithm has efficient processing capabilities and good generalization performance. Therefore, it is particularly suitable for processing high-dimensional feature data such as driver data and vehicle data. In addition, XGBoost is often used to build powerful prediction models. It can integrate multiple feature information to improve the accuracy of road hypnosis recognition. The structure of XGBoost is shown in Figure 2.

#### 2.2.3. LIME-SHAP Method

LIME (Local Interpretable Model-Agnostic Explanations) is a model interpretation technique that provides interpretable local explanations for complex machine learning models. The core idea is that a simple linear model is constructed for the local region of a specific sample to explain its prediction outcome. The algorithm generates a neighborhood dataset by applying small perturbations to the model inputs for a target sample point. A simple linear model is then fitted within this neighborhood to analyze the contribution of specific features to the prediction outcome. LIME can decompose the predictions of complex models into the weighted contributions of each feature [38,39,40]. SHAP (Shapley additive explanations) is a model interpretation method based on game theory. It is used to quantify the global and local impact of each feature on model predictions. Its core is based on Shapley values, which assign a clear contribution value to each feature. The additive feature model is used by SHAP to calculate the Shapley values for each feature. The Shapley values ensure fairness in the explanation by considering the average marginal contribution of all feature combinations. The global importance and local contribution of each feature to the prediction result can be provided by SHAP [41,42,43].

### 2.3. Experiment

#### 2.3.1. Participants

In the selection of drivers, participants who are more prone to road hypnosis were chosen for the experiment. Due to the cautious driving behavior of novice drivers, road hypnosis is less likely to occur. Therefore, drivers with at least 8 years of driving experience were selected. The age range of the drivers was set between 26 and 60 years. Additionally, drivers were required to be in good physical and mental health, with relatively stable sleep habits and consistent sleep schedules. In addition, to facilitate the collection of road hypnosis states in non-monotonous road environments, the recruited participants were required to have fixed daily commuting routes. A total of 30 participants were recruited, with a male-to-female ratio of 8:2.

#### 2.3.2. Experiment Route

**Figure 3:**
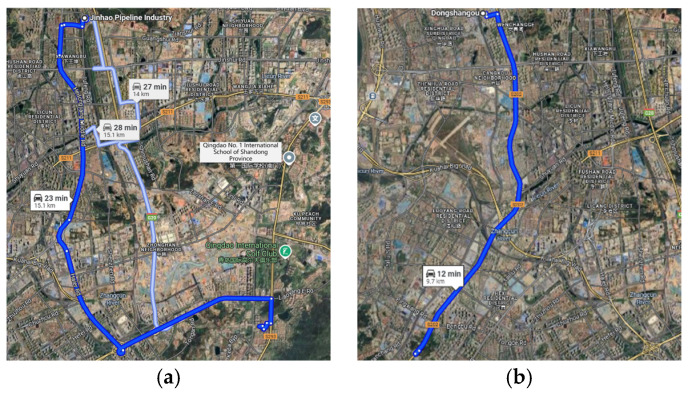
Experiment Routes. (**a**) Driver A. (**b**) Driver B.

Most prior studies were conducted on fixed driving routes in monotonous driving environments, such as highways or tunnels, which are prone to inducing road hypnosis states [35,36,37,38,39]. In this study, driver and vehicle data were collected under various scenarios to construct a dataset for non-fixed driving routes. Road hypnosis states were further identified. The participants in this study were selected through social recruitment. Each participant had a distinct, fixed daily commuting route. Therefore, for each participant, a specific driving route was selected for the experiment. This route was the same as their regular commuting route. The experiment was conducted during a time period similar to their usual commuting time. As shown in Figure 3, Driver A’s daily fixed commuting route is from the XX community in Shibei District, Qingdao, to the Laoshan campus of Qingdao University of Science and Technology in Laoshan District, Qingdao. Driver B’s daily fixed commuting route is from the XX community in Licang District, Qingdao, to Qingdao Haier Co., Ltd. in Laoshan District, Qingdao. Both routes involve complex and variable urban roads with significant curvature changes. The routes pass through locations such as schools, CBD business districts, and companies. This results in a dynamic and varied scene. The specific method for collecting road hypnosis data in the experiment can be found in references [26,27,28,29,30].

#### 2.3.3. Scene and Equipment

**Figure 4:**
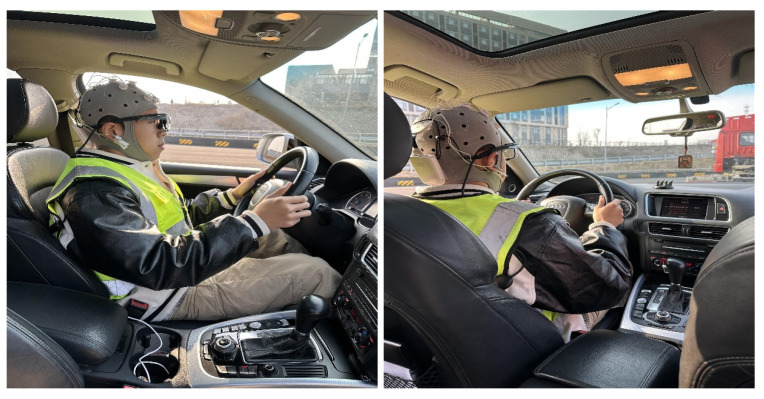
Experimental environment.

Vehicle driving experiments were conducted in this study. The experimental platform consisted primarily of a comprehensive road test vehicle, a laptop, and a dashcam. The environment for the vehicle driving experiment is shown in Figure 4.

**Figure 5:**
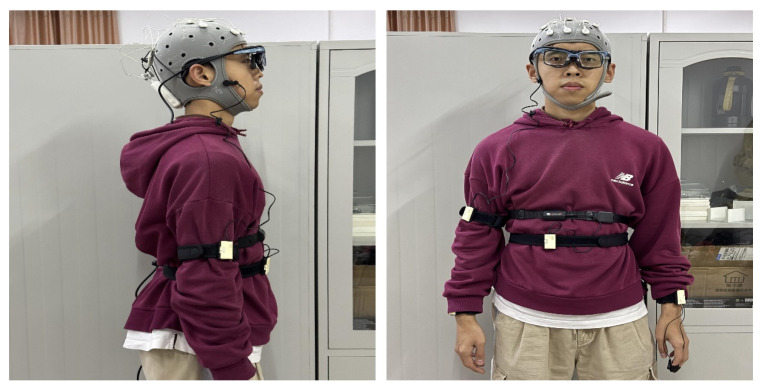
Image showing how the device is worn.

The eye movement device used was the aSee Glasses from Beijing Qixin Yiwei Company (Beijing, China). Full experimental functions are provided. These functions consist of eye movement data recording, analysis and visualization, and data export. The EEG device used was the Enobio Dx, developed by Yingfu Instrument Technology (Shanghai) Co., Ltd. (Shanghai, China). This device restores the signal-to-noise ratio of the raw EEG signals, which allows the perfect integration of the signal’s high dynamic range. It records all DC signals accurately and removes artifacts. The vehicle data collection device was independently developed by the research team. It is based on Gaode Navigation and only requires a mobile phone to collect real-time dynamic parameters of the vehicle during driving [44]. The overall installation of the device is shown in Figure 5.

#### 2.3.4. Procedure

**Figure 6:**
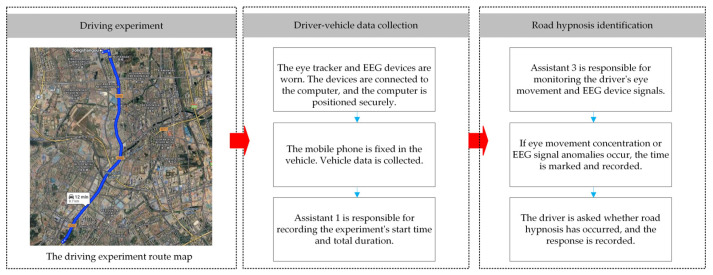
Experimental framework and process.

The main objective of this experiment is to collect EEG, eye movement, and vehicle data from drivers during normal commutes along fixed routes. In addition to the participants, three assistants were involved in the experiment. The experiment was divided into three main parts: the driving experiment, driver–vehicle data collection, and road hypnosis identification. One assistant observed traffic flow and road conditions to ensure safety. The driver wearing the experimental equipment may have an impact on the driver. Therefore, the driver will be required to wear the experimental equipment for a period of time before the experiment to reduce the impact on the experimental results. The experimental framework and process are shown in Figure 6.

## 3. Road Hypnosis Identification Model

**Figure 7:**
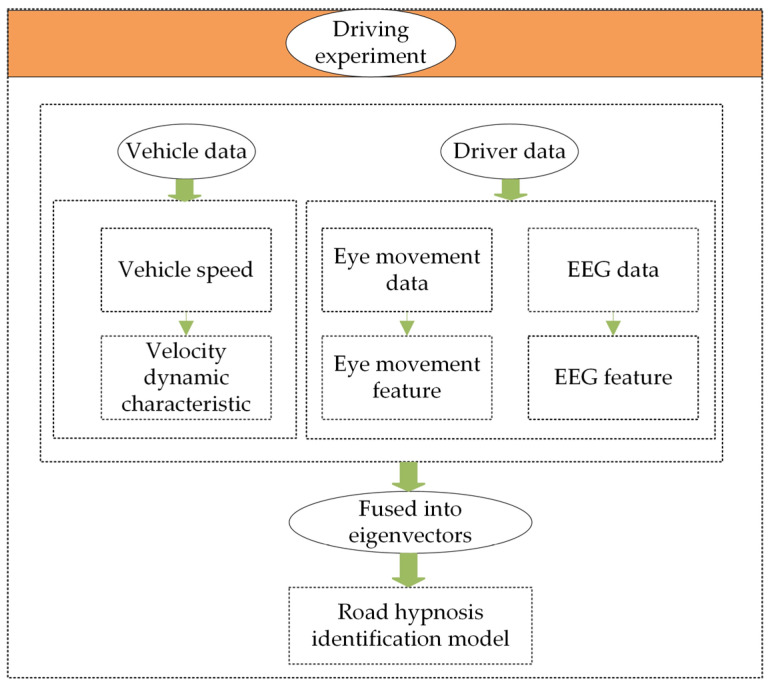
Road hypnosis identification model.

After the data collection is completed, the obtained data are organized, and 30 sets of vehicle driving experiment data are obtained. Each dataset is screened and rated based on the characteristics of the experimental videos and the data itself. The validity of the screened data is then confirmed. Four datasets are excluded due to driver distraction and road rage caused by complex traffic conditions during driving. After filtering, 26 complete and valid real-vehicle driving datasets are obtained. In the final obtained experimental data, EEG data and eye movement data are preprocessed. The power spectral density (PSD) method, local linear embedding (LLE) method, and point-by-point calculation method are used to extract features from EEG data, eye movement data, and vehicle data, respectively. Timestamp synchronization is applied to different modalities to ensure that multi-source data are accurately recorded under the same time reference. Based on this, EEG data, eye movement data, and vehicle data are normalized into a unified feature vector. This feature vector is then used as the input data for the XGBoost-HMM algorithm to construct the road hypnosis state identification model. The detailed model construction framework is shown in Figure 7.

### 3.1. Driver Data Preprocessing

(1) EEG Data Preprocessing

Due to the difficulty in directly labeling EEG signals, the abnormal fixation points in the eye movement video and the time points when the experimental drivers are actively questioned are selected as the criteria for labeling the EEG signals in this study. First, the eye movement data file is imported, and the data containing the time periods of “road hypnosis onset” and “road hypnosis cessation” are extracted. Next, the data within these time periods are cleaned, and invalid data are removed to ensure that each valid time has a clear start and end time. Subsequently, the EEG data are preprocessed by removing unnecessary channels, and the data are re-referenced to the average reference. A bandpass filter (0.2–40 Hz) is applied to remove noise and retain the signals associated with road hypnosis. The filtering process is as follows:

1. Design the frequency response of the filter.

The low pass filter is as follows:

(1) $$H_{low}\left(f\right)=\left\{\begin{array}{l} 0,\;\mathrm{if}\;f\leq 40\mathrm{Hz}\\ 1,\;\mathrm{if}\;f>40\mathrm{Hz} \end{array}\right.$$

In this case, $H_{low}\left(f\right)$ is the frequency response function of the low-pass filter. 40 Hz is the cutoff frequency of the low-pass filter.

The high pass filter is as follows:

(2) $$H_{high}\left(f\right)=\left\{\begin{array}{l} 0,\;\mathrm{if}\;f<0.5\mathrm{Hz}\\ 1,\;\mathrm{if}\;f\geq 0.5\mathrm{Hz} \end{array}\right.$$

In this case, $H_{high}\left(f\right)$ is the frequency response function of the high-pass filter. 0.5 Hz is the cutoff frequency of the low-pass filter.

The frequency response of the bandpass filter is:

(3) $$H\left(f\right)=H_{low}\left(f\right)\cdot H_{high}\left(f\right)$$

In this case, $H\left(f\right)$ is the overall frequency response of the bandpass filter.

2. Convert the signal to the frequency domain.

Fast Fourier transform (FFT) is used to convert EEG signals from the time domain to the frequency domain.

(4) $$X\left(f\right)=FFT\left(x\left(t\right)\right)$$

In this case, $X\left(f\right)$ is the representation of the input signal $x\left(t\right)$ in the frequency domain.

3. Apply the filter.

Multiply the frequency response $X\left(f\right)$ of the signal by the frequency response $H\left(f\right)$ of the bandpass filter.

(5) $$Y\left(f\right)=X\left(f\right)\cdot H\left(f\right)$$

In this case, $Y\left(f\right)$ is the filtered signal.

4. Convert the signal back to the time domain.

The Inverse Fast Fourier Transform (IFFT) is used to convert the filtered signal Y(f) back to the time domain.

(6) $$y\left(t\right)=IFFT\left(Y\left(f\right)\right)$$

In this case, $y\left(t\right)$ is the filtered time domain signal. It is the final output signal.

The corresponding EEG signal segments are extracted from the EEG data based on the time periods obtained from the eye movement data file. Each time is cropped to ensure that the data fall within the valid range of EEG collection. The segments are then concatenated into a new EEG data object. Finally, a sliding window method is applied for frequency band analysis of the EEG signals. The window length is set to 0.5 s, with a step size of one sampling point. The power spectral density (PSD) of the signals within each window is calculated with the Welch method. The average power for each frequency band (δ-wave, θ-wave, α-wave, β-wave, γ-wave) is calculated. The specific calculation process is as follows:

- Segmentation and windowing

The signal $X_{i}$ for each window is segmented, with the segmented signal denoted as $x_{m}(n)$. Each segment has a length of $N_{s}$ (defaulting to the entire window length M) and a window function $w\left(n\right)$ is applied. The specific formula is as follows:

(7) $$x_{m}^{\left(w\right)}\left(n\right)=x_{m}\left(n\right)\cdot w\left(n\right),\;n=0,\;1,\;\ldots ,\;N_{s}-1$$

b. Fast Fourier Transform (FFT)

The FFT is applied to the windowed signal. The specific formula is as follows:

(8) $$X_{m}\left(f\right)=FFT\left\{x_{m}^{\left(w\right)}\left(n\right)\right\}$$

c. Power Spectrum Calculation

The power spectrum of each segment is calculated as follows:

(9) $$P_{m}\left(f\right)=\frac{{\left|X_{m}\left(f\right)\right|}^{2}}{N_{s}\cdot norm\left(w\right)}$$

In this case, $norm\left(w\right)$ is the normalization factor of the window function.

d. Band power averaging

The power spectra of all segments are averaged to obtain the PSD estimate of window $X_{i}$. The specific formula is as follows:

(10) $$P_{i}\left(f\right)=\frac{1}{M_{s}}\sum_{m=1}^{M_{s}}P_{m}\left(f\right)$$

In this case, $M_{s}$ is the number of segments.

e. Frequency band power calculation

The PSD estimate $P_{i}\left(f\right)$ is divided into the following frequency bands based on the frequency range: δ (Delta): 0.5–4 Hz, θ (Theta): 4–8 Hz, α (Alpha): 8–12 Hz, β (Beta): 12–30 Hz, γ (Gamma): 30–40 Hz. The average power for each frequency band is:

(11) $$P_{band}=\int_{f_{1}}^{f_{2}}P_{i}\left(f\right)df\approx \sum_{f=f_{1}}^{f_{2}}P_{i}\left(f\right)\cdot \Delta f$$

In this case, $f_{1}$ and $f_{2}$ are the starting and ending frequencies of the frequency band, Δf is the frequency resolution.

(2) Eye movement data preprocessing.

First, the eye movement data are cleaned by removing invalid and outlier values collected during the experiment. Second, road hypnosis is typically characterized by slow changes or reduced fluctuations in pupil diameter and fixation points. To eliminate noise interference and extract key signal features, an FIR (Finite Impulse Response) filter is applied to preprocess the eye movement data.

The FIR filter is a commonly used digital filter. Its stability and linear phase characteristics ensure effective noise elimination and key signal feature extraction. The authenticity and integrity of the eye movement data along the time axis are also preserved. First, a low-pass filter is designed to remove high-frequency noise and retain the low-frequency components of the eye movement signal, such as the smooth changes in pupil diameter and trend changes in fixation points. At the same time, a high-pass filter is designed to eliminate signal drift caused by light changes. Finally, the sliding average method is applied to smooth the eye movement data, which reduces the impact of random fluctuations on subsequent feature extraction. The basic formula of the FIR filter is as follows:

(12) $$y[n]=\sum_{k=0}^{M-1}h[k]\cdot x[n-k]$$

In this case, y[n] is the filtered eye movement signal, x[n] is the raw eye movement signal, h[k] is the impulse response coefficient of the low-pass FIR filter, M is the filter order, and n−k is the delay of the input signal.

The FIR filter is designed with the window function method. The impulse response of the ideal filter is as follows:

(13) $$h_{i}[k]=\left\{\begin{array}{l} (-)\sin(2\pi f_{c}(k-\frac{M}{2})),\;k\neq \frac{M}{2}\\ 2f_{c},\;k=\frac{M}{2} \end{array}\right.$$

In this case, $f_{c}$ is the cutoff frequency, $f_{s}$ is the sampling rate.

The window function is applied to the impulse response to reduce truncation errors:

(14) $$h[k]=h_{i}[k]\cdot w[k]$$

The sliding window is set to 20 sample points. The specific formula for the sliding average is as follows:

(15) $$H[n]=\frac{1}{N}\sum_{k=0}^{N-1}y[n-k]$$

In this case, H[n] is the smoothed eye movement signal, N is the sliding window, n is the index of the current sampling point, k is the offset of the point within the sliding window. See Table 1.

Finally, key features related to road hypnosis are extracted from the preprocessed eye movement data with the Local Linear Embedding (LLE) method. The LLE method performs feature extraction based on the local geometric structure of the data points. LLE performs dimensionality reduction by preserving the local adjacency relationships within the eye movement data. This process captures the nonlinear structure of the data. The specific process is as follows:

- The adjacency graph is constructed.

For each data point $x_{i}$, the distance to all other data points $x_{j}$ is calculated with the Euclidean distance.

(16) $$d_{\mathrm{ij}}={\left\|x_{i}-x_{j}\right\|}_{2}$$

In this case, ${\left\|x_{i}-x_{j}\right\|}_{2}$ represents the Euclidean distance between points $x_{i}$ and $x_{j}$.

The K nearest points to $x_{i}$ are selected as the neighbors $N_{i}$.

(17) $$N_{i}=\left\{x_{j}|j\in \left\{1,\;2,\;\ldots ,\;n\right\}\;j\neq i\right\}$$

b. Reconstruction weights are calculated.

The weight $w_{ij}\left(j\in N_{i}\right)$ for each data point $x_{i}$ is calculated, so that the data point can be reconstructed through its neighboring points $x_{j}$.

(18) $$x_{i}=\sum_{j\in N_{i}}w_{ij}x_{j}$$

In this case, $w_{ij}$ is the coefficient of the linear combination.

c. The weight matrix is constructed.

The reconstruction weights $w_{ij}$ of all data points are summarized into a weight matrix W, which records the local relationships between all data points. When constructing the weight matrix, it is necessary to ensure that the matrix is symmetric:

(19) $$W_{ij}=W_{ji}$$

d. The optimization problem is solved.

The following objective function is minimized to obtain the low-dimensional embedding of the data points:

(20) $$\underset{Y}{\min}\sum_{i}\left(y_{i}-\sum_{j\in N_{i}}w_{ij}y_{j}\right)$$

In this case, $Y=[y_{1},\;y_{2},\;\ldots ,\;y_{n}]$ is the coordinate matrix of the low-dimensional embedding, where each $y_{i}$ represents the coordinates of the corresponding data point $x_{i}$ in the low-dimensional space.

To preserve the local neighborhood relationships of the data points in the low-dimensional embedding Y, the objective function can be transformed into an eigenvalue problem:

(21) $$Y^{T}{\left(I-W\right)}^{T}(I-W)Y=\lambda Y^{T}Y$$

In this case, I is the identity matrix, W is the weight matrix, λ are the eigenvalues.

The representation of the data points in the low-dimensional space Y is obtained by solving the eigenvalue problem.

According to the above steps, LLE maps the high-dimensional eye movement data to a low-dimensional space, and the important features from the eye movement data are extracted.

### 3.2. Vehicle Data Preprocessing

The processing of driving behavior data requires synchronization of the timestamps with the EEG signals and eye movement data, as well as the cleaning of missing and outlier values. Based on this, dynamic features are extracted for the vehicle’s speed and acceleration with sliding window analysis and point-by-point calculation. Each variable column is maintained as a time series after feature extraction. The features extracted from speed include the sliding average speed and speed fluctuation, which reflect the local speed change trend. The features extracted from acceleration include the sliding average acceleration and acceleration rate of change, which capture rapid acceleration or deceleration behaviors. The specific processing steps are as follows:

(1) Sliding average speed

(22) $$AvgSpeed_{wi}=\frac{1}{T_{w}}\int_{t_{i}}^{t_{i}+T_{w}}Speed\left(t\right)dt$$

In this case, $AvgSpeed_{wi}$ is the average speed within the window $w_{i}$, $T_{w}$ is the length of the sliding window, $t_{i}$ is the starting time of the window, and $Speed\left(t\right)$ is the instantaneous speed at time t.

(2) Speed fluctuation (standard deviation)

(23) $$StdSpeed_{wi}=\sqrt{\frac{1}{N}\sum_{j=1}^{N}{\left(Speed_{j}-AvgSpeed_{wi}\right)}^{2}}$$

In this case, $StdSpeed_{wi}$ is the standard deviation of speed within window $w_{i}$, N is the number of sampling points within the window, $Speed_{j}$ is the speed at the j time point within the window.

(3) Sliding average acceleration

(24) $$AvgAcceleration_{wi}=\frac{1}{T_{w}}\int_{t_{i}}^{t_{i}+T_{w}}Acceleration(t)dt$$

In this case, $AvgAcceleration_{wi}$ is the average acceleration within window $w_{i}$, Acceleration(t) is the instantaneous acceleration at time t, $T_{w}$ is the length of the sliding window, $t_{i}$ is the starting time of the window.

(4) Acceleration rate of change

(25) $$Jerk_{i}=\frac{Acceleration_{i+1}-Acceleration_{i}}{t_{i+1}-t_{i}}$$

In this case, $Jerk_{i}$ is the acceleration rate of change at the i−th time point, $Acceleration_{i}$ is the acceleration at the i−th time point, $t_{i}$ is the i−th time point.

### 3.3. Feature Normalization

After extracting the key features of the driver and vehicle data, the features are concatenated to form a unified feature space. This method balances the differences in multimodal data and explains the contribution of each feature to the model through feature importance analysis with the XGBoost algorithm. The specific processing steps are as follows:

(1) Feature normalization

Standardization is applied to balance the differences in feature dimensions. All features are given a mean of 0 and a variance of 1.

(26) $$f_{std}=\frac{f-\mu }{\sigma }$$

In this case, f is the original feature value, μ is the mean of the feature value, σ is the standard deviation of the feature.

(2) Feature alignment

The sampling frequencies of different modalities are inconsistent. The time window method is applied to align the features.

(27) $$f_{aligned.i}^{\left(k\right)}=\frac{1}{T}\sum_{t=k-T+1}^{k}f_{i}^{\left(t\right)}$$

In this case, T is the time window, k is the current time point of the data, t represents the time point traversal variable, and $f_{i}^{\left(t\right)}$ is the i−th original feature value at the t−th time point.

### 3.4. Model Construction, Calibration, and Verification

HMM has a natural advantage in sequential modeling. It effectively captures the transition process of system states. It is particularly suitable for describing the relationship between latent states such as normal driving and road hypnosis driving, and observed data such as EEG, eye movement, and vehicle acceleration. In the road hypnosis identification problem, the driver’s state changes continuously, and these states are often not directly observable. The HMM can effectively infer the latent hidden states through the observed data. For example, during driving, the driver’s state may transition from alertness to drowsiness, and then to deep hypnosis. The HMM models this state transition process naturally by setting state transition probabilities. It is well suited for road hypnosis identification tasks. The traditional HMM assumes that the observed data follow a Gaussian distribution. However, real-world data often exhibit complex nonlinear relationships. When handling multimodal data, the relationships between features are typically nonlinear and involve complex interactions. XGBoost is a powerful gradient boosting decision tree algorithm. It automatically learns nonlinear patterns in the data to provide more accurate predictions. Therefore, the XGBoost algorithm is chosen to optimize the observation model of the HMM. This enables the model to better handle complex data features. The specific optimization process is as follows:

(1) Basic HMM structure

a. The hidden state transition matrix A: The transition probabilities of the system’s states at different time points are described.

(28) $$a_{ij}=P\left(S_{t}=S_{j}|S_{t-1}=S_{i}\right)$$

In this case, $S_{t}$ is the hidden state at time t, $a_{ij}$ is the probability of transitioning from state $S_{i}$ to state $S_{j}$.

b. The observation probability matrix B: The matrix describes the probability of the hidden state $S_{t}$ given the observation $O_{t}$. For the traditional HMM, it is typically assumed that the observation $O_{t}$ comes from a certain probability distribution.

(29) $$b_{j}\left(o_{t}\right)=P\left(O_{t}=o_{t}|S_{t}=S_{j}\right)$$

In this case, $b_{j}\left(o_{t}\right)$ is the probability of generating observation $o_{t}$ in state $S_{j}$, $o_{t}$ is the observation data at time t.

(2) XGBoost training process

The XGBoost algorithm is used to learn the observation probability $b_{j}\left(o_{t}\right)$ in the HMM algorithm, which represents the probability of observing $O_{t}$ given the hidden state $S_{j}$.

a. The observation data $O_{t}$, along with EEG, eye movement, and vehicle features, are extracted under the hidden state. These features $X_{t}$ are used as the model input, with $y_{t}$ representing the corresponding prediction probability.

b. For each hidden state $S_{j}$, an XGBoost model is trained. The conditional probability $b_{j}\left(o_{t}\right)$ of the observation $O_{t}$ given the feature $X_{t}$ is learned. The objective function of XGBoost is as follows:

(30) $$L\left(\theta \right)={\sum_{t=1}^{T}\left({\overset{\Lambda }{b}}_{j}\left(o_{t}\right)-b_{j}\left(o_{t}\right)\right)}^{2}+\Omega \left(\theta \right)$$

In this case, ${\overset{\Lambda }{b}}_{j}\left(o_{t}\right)$ is the observation probability predicted by the XGBoost model, $b_{j}\left(o_{t}\right)$ is the actual observed probability, $\Omega \left(\theta \right)$ is the regularization term, which prevents overfitting.

c. The model parameters θ are obtained by optimizing the objective function $L\left(\theta \right)$ with the training set $\left\{X_{t},\;y_{t}\right\}$.

d. After the model is trained, the XGBoost model is used to predict each hidden state $S_{j}$ and output the predicted probability ${\overset{\Lambda }{b}}_{j}\left(o_{t}\right)$ for the observation data $O_{t}$. These predicted observation probabilities ${\overset{\Lambda }{b}}_{j}\left(o_{t}\right)$ replace the observation probability $b_{j}\left(o_{t}\right)$ in the traditional HMM.

(3) Integrating XGBoost with HMM

a. The forward algorithm: The posterior probability of the hidden states is calculated given the observation sequence.

(31) $$\alpha_{t}\left(i\right)=P\left(O_{i},\;O_{2},\;\ldots ,\;O_{t},\;S_{t}=S_{i}\right)$$

In this case, $\alpha_{t}\left(i\right)$ is the probability that the system is in state $S_{i}$ at time t and observes $O_{i},\;O_{2},\;\ldots ,\;O_{t}$.

The optimized formula is as follows:

(32) $$\alpha_{t}\left(j\right)=\sum_{i=1}^{N}\alpha_{t-1}\left(i\right)a_{ij}\overset{\Lambda }{b}\left(o_{t}\right)$$

In this case, $a_{ij}$ is the hidden state transition probability.

b. Viterbi algorithm: the most probable hidden state sequence is found.

(33) $$\delta_{t}\left(i\right)=\underset{S_{1},\;S_{2},\;\ldots ,\;S_{t-1}}{\max}\left(\alpha_{t-1}\left(i\right)a_{ij}b_{j}\left(o_{t}\right)\right)$$

In this case, $\delta_{t}\left(i\right)$ is the probability of the most probable hidden state sequence at time t through the path of state $S_{i}$.

The forward algorithm or Viterbi algorithm is used in combination with the observation probability ${\overset{\Lambda }{b}}_{j}\left(o_{t}\right)$ optimized by XGBoost and the state transition matrix A. The most probable hidden state sequence $S=\{S_{1},\;S_{2},\;\ldots ,\;S_{T}\}$ is then inferred.

This study constructs a road hypnosis identification model by combining the XGBoost algorithm with the Hidden Markov algorithm. The Platt scaling method is applied to calibrate the model outputs. This increases the accuracy of the predictions. To further validate the model, K-fold cross-validation is used. This method randomly divides the dataset into K subsets. In each iteration, K − 1 subsets are used for training, and the remaining subset is used for validation. This process is repeated K times where each subset serves as the validation set once. K-fold cross-validation ensures that all results are used for both training and testing. Each result is used once for both, which allows for a better evaluation of the model’s performance.

## 4. Results and Discussion

**Figure 8:**
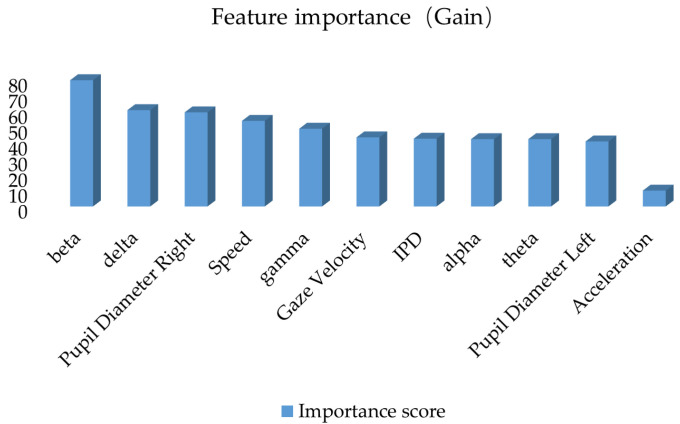
Feature importance ranking based on the gain metric.

**Figure 9:**
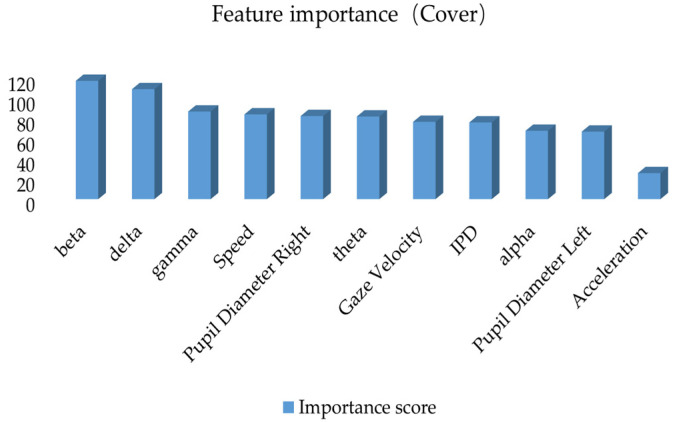
Feature importance ranking based on the cover metric.

**Figure 10:**
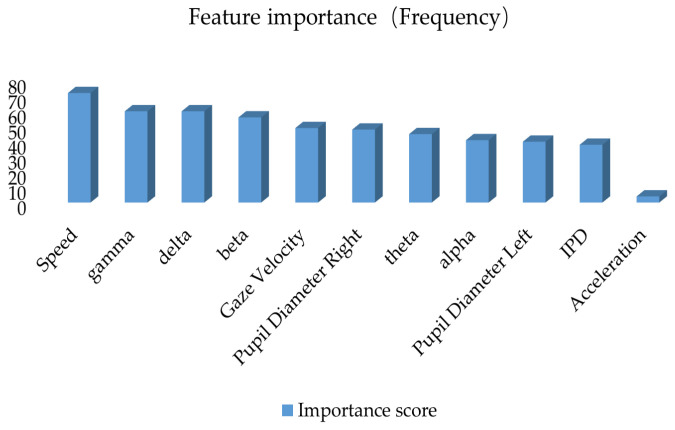
Feature importance ranking based on the frequency metric.

This study constructs a road hypnosis state identification model by integrating driver data and vehicle data, with XGBoost optimization of the HMM algorithm. The gain from XGBoost is used to measure the average contribution of each feature to the improvement of the model’s prediction performance. Coverage is used to measure the proportion of data samples covered by each feature during splitting. Frequency is used to measure the number of times each feature is used across all split nodes. The experimental results are shown in Figure 8, Figure 9 and Figure 10.

As shown in Figure 8, when gain is used as the evaluation metric, the beta wave has the highest gain value. This indicates that this feature significantly improves the model’s prediction performance at the split nodes and is the most critical feature for identifying road hypnosis states. The next in ranking are the delta wave, pupil diameter right, speed, gamma, gaze velocity, inter-pupillary distance (IPD), alpha wave, theta wave, and pupil diameter left. This indicates that the integration of eye movement data, vehicle data, and EEG data plays a significant role in the overall identification of road hypnosis. The low gain for acceleration suggests that this feature has a minor impact on improving the accuracy of the road hypnosis identification model and contributes only to certain samples.

As shown in Figure 9, when coverage is used as the evaluation metric, the beta wave still has the highest gain value. This indicates that it not only improves the model’s performance but is also applied to more data samples. It is a key feature for distinguishing road hypnosis. This suggests that these features play an important role in the broad applicability of the model. The coverage for pupil diameter right, theta wave, gaze velocity, IPD, alpha wave, theta wave, and pupil diameter left is relatively high. This further indicates that the data fusion method can effectively identify road hypnosis. The low coverage for acceleration suggests that these variables may only have an effect on specific samples and have minimal impact on the final accuracy of road hypnosis identification.

As shown in Figure 10, when frequency is used as the evaluation metric, speed is the most frequently used feature. This indicates that the feature is frequently used at the split nodes of the model and is an important fundamental feature in the model. Next in rank are gamma, delta, and beta waves, pupil diameter right, theta wave, gaze velocity, IPD, alpha wave, theta wave, and pupil diameter left. This suggests that these features may not contribute the most in a single split but are frequently used throughout the overall splitting process.

The beta waves rank high in both gain and coverage. However, its frequency of use is slightly lower. This is because it is more suitable for splitting at key local nodes rather than for high-frequency features. The frequency of acceleration’s use is relatively low. This indicates that these variables are selected less frequently at the split nodes and may have a minimal impact on the accuracy of road hypnosis identification.

A comprehensive analysis of these three types of results shows that, whether using gain, coverage, or frequency metrics, beta and delta (EEG features) consistently rank highly in the importance evaluation. This indicates that EEG features are crucial for identifying road hypnosis states. They significantly enhance model performance and are applicable to a wide range of samples. Pupil diameter right (right pupil diameter) and speed show high importance in gain, coverage, and frequency metrics. This suggests that the integration of eye movement data and vehicle data significantly improves the model’s identification ability. High-gain features such as beta and delta primarily improve single-split performance. High-coverage features such as gamma and speed ensure the broad applicability of features. High-frequency features such as speed and gamma hold an important position in overall decision-making. These features complement each other and create a comprehensive road hypnosis identification model. Some features, such as acceleration, score low in all three metrics. These may be secondary or redundant features and can be removed in model optimization studies.

**Figure 11:**
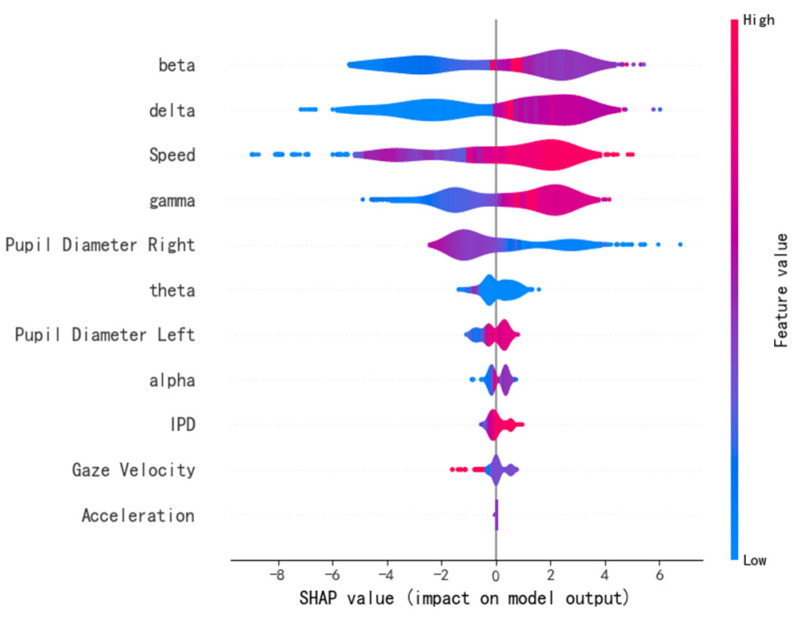
SHAP feature importance distribution.

**Figure 12:**
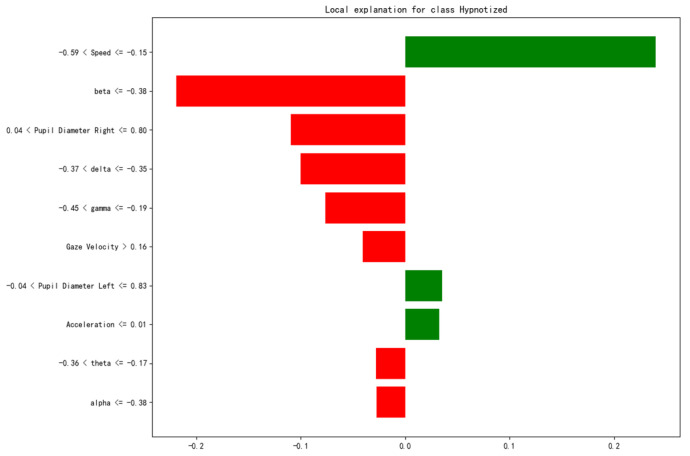
LIME single-sample feature contribution.

In addition, SHAP and LIME were used to further reveal the internal decision-making mechanism of the model. The contribution of each feature to the model’s predictions was quantified from both global and local perspectives. This further validated the key role of each feature in identifying road hypnosis. The results are shown in Figure 11 and Figure 12.

In Figure 11, each row represents a feature. Each point represents the SHAP value of a sample. The *x*-axis shows the magnitude of the SHAP value, which reflects the positive or negative contribution of the feature to the model output. The color represents the feature value, with low values shown in blue and high values shown in red.

It can be observed from the figure that the beta wave feature has the most significant impact on the model output. High values of the beta wave (red) typically positively influence the model’s prediction of road hypnosis, while low values (blue) have a negative impact. This indicates that the intensity variation of the beta wave is an important indicator for determining the driver’s hypnotic state. Similarly, high values of the delta wave, speed, and gamma wave make significant positive contributions to the model output. This indicates that changes in vehicle speed and the intensity of the gamma and delta waves are closely related to the identification of road hypnosis. The SHAP values of pupil diameter left, theta wave, gaze velocity, alpha wave, IPD, and pupil diameter right are more concentrated. This indicates that the integration of eye movement data and vehicle data plays an important role in the overall identification of the road hypnosis model. The SHAP values of acceleration have a narrow distribution and have a small impact on the model’s prediction. This suggests that this variable may have a minimal effect on road hypnosis identification.

In Figure 12, the *x*-axis represents the magnitude of the feature’s contribution to the model output. The red bars represent the negative impact of the feature on predicting a non-hypnotic state, while the green bars represent the positive impact on predicting a hypnotic state.

From Figure 12, speed is the feature with the greatest contribution to the classification result. Its range (−0.59, −0.15) makes a significant positive contribution to the model’s prediction (green bars). This suggests that within this speed range, the model is more likely to predict the driver is in a “hypnotic state”. Acceleration also provides some positive support for the prediction of this sample, but its impact is minimal. Beta wave and pupil diameter right are the features with the greatest negative contribution. This indicates that for this sample, the intensity of the beta waves and the pupil diameter right reduced the probability of the model predicting a “hypnotic state”. The alpha wave, gamma wave, theta wave, delta wave, and gaze velocity features make the next largest negative contributions to the model’s prediction. However, they still have a certain level of influence.

A comprehensive analysis of these two types of results shows that the SHAP plot displays the impact and direction of each feature on the prediction across the global scope.

The beta wave, delta wave, speed, and gamma wave are key features of the model. They significantly improve the global predictive ability of the road hypnosis identification model. The contributions of eye movement and vehicle data, such as pupil diameter right and speed, are relatively concentrated. The LIME plot focuses on the feature contributions of individual samples. It shows that variables such as speed, beta wave, and pupil diameter right are linked to the road hypnosis identification model. Consistent with the global trend observed in SHAP, beta wave, speed, and delta wave remain important features for local predictions. However, in specific samples, the positive influence of certain features, such as beta wave, is greater.

**Figure 13:**
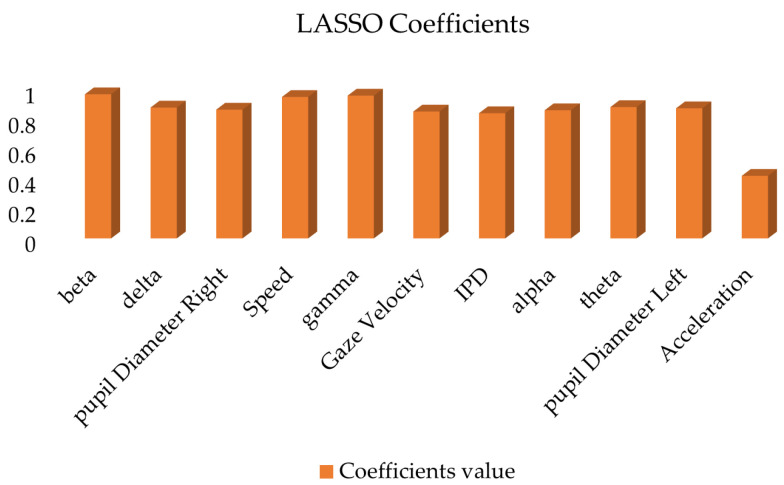
LASSO coefficient value.

**Figure 14:**
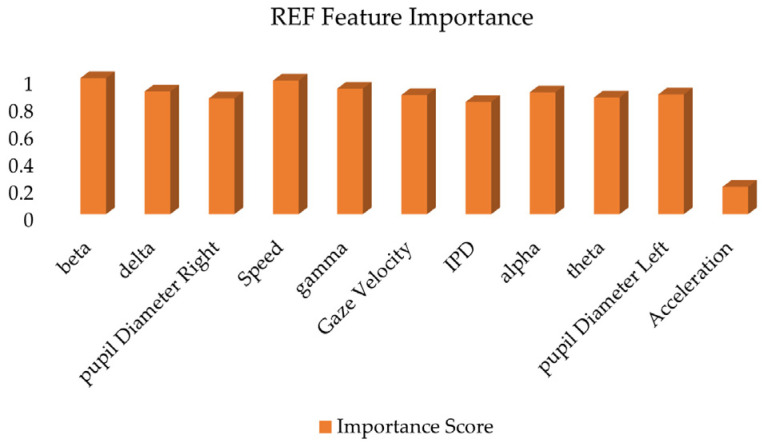
REF feature importance score.

In order to further verify the results of feature importance analysis. Least Absolute Shrinkage and Selection Operator (LASSO) and recursive feature elimination (RFE) feature selection methods were introduced. LASSO regression is a regression method that introduces L1 regularization. In LASSO regression, the model imposes an L1 penalty on the coefficients of the features. The coefficients of some features are forced to become zero. Feature selection is then achieved. In this way, LASSO can automatically eliminate features that have little impact on the road hypnosis recognition model. Retain the most predictive features. It is particularly suitable for processing high-dimensional data (such as driver data and vehicle data). Recursive feature elimination is a recursive feature selection method. RFE gradually reduces the dimensions of feature sets (such as driver data and vehicle data) through multiple iterations. Until the most important features are left. This method recursively eliminates redundant features so that the final feature set can provide the greatest improvement to the performance of the model. The results are shown in Figure 13 and Figure 14.

The driver data and vehicle data are selected by LASSO regression and RFE, respectively. The results show that beta wave, delta wave, pupil diameter right, speed, gamma wave, gaze velocity and other features are considered important in both methods. They have higher coefficient values and importance scores. Acceleration scores are lower in both methods. Therefore, it is considered to contribute less to the model and can be eliminated from the final model.

Based on the feature importance analysis of driver and vehicle data, in the driver data, the delta, theta, alpha, beta, and gamma waves from EEG data and the gaze velocity, left and right pupil sizes, and interpapillary distance from eye movement data all contribute to determining whether the driver is in a road hypnosis state. In the vehicle data, vehicle speed contributes to determining whether the driver is in a road hypnosis state. Therefore, the data that significantly contribute to determining the road hypnosis state and the corresponding environmental data are used as input data to construct the road hypnosis identification model based on the Hidden Markov Model. The regression coefficients and intercept values output by the constructed model are shown in Table 2.

The value of the intercept $\beta_{0}$ is 0.471. The values of $\beta_{2}$, $\beta_{3}$, $\beta_{11}$ and $\beta_{13}$ are all 0.

Therefore, the obtained road hypnosis judgment calculation formula is as follows:

(34) $$\mathrm{Hypnosis}\;\mathrm{Degree}=\sum_{i=1}^{14}x_{i}\beta_{i}+0.471$$

This study introduces the following evaluation metrics to assess the model’s performance.

Mean squared error (MSE) is used to evaluate the model’s prediction error. Its calculation formula is as follows:

(35) $$MSE=\frac{1}{n}{\sum_{i=1}^{n}\left(y_{i}-{\overset{\wedge }{y}}_{i}\right)}^{2}$$

In this case, $y_{i}$ is the true value of the road hypnosis state, ${\overset{\wedge }{y}}_{i}$ is the predicted value of the road hypnosis state, n is the number of samples.

The coefficient of determination (R^2^) is used to measure the extent to which the model fits the data. Its calculation formula is as follows:

(36) $$R^{2}=1-\frac{\sum_{i=1}^{n}{\left(y_{i}-{\overset{\wedge }{y}}_{i}\right)}^{2}}{\sum_{i=1}^{n}{\left(y_{i}-{\overset{-}{y}}_{i}\right)}^{2}}$$

In this case, $y_{i}$ is the mean of the true values of the road hypnosis state.

Root mean squared error (RMSE) is the square root of MSE. Its magnitude is the same as that of the true values. Its calculation formula is as follows:

(37) $$RMSE=\sqrt{\frac{1}{n}{\sum_{i=1}^{n}\left(y_{i}-{\overset{\wedge }{y}}_{i}\right)}^{2}}$$

Mean absolute error (MAE) calculates the average of the absolute differences between the predicted values and the true values. Its calculation formula is as follows:

(38) $$MAE=\frac{1}{n}\sum_{i=1}^{n}\left|y_{i}-\overset{\wedge }{y_{i}}\right|$$

Explained variance (EV) represents the proportion of the target variable’s variance that can be explained by the model. Its value ranges from 0 to 1. The closer the EV value is to 1, the stronger the model’s explanatory power for the target variable. Its calculation formula is as follows:

(39) $$EV=1-\frac{Var(y-\overset{\wedge }{y})}{Var(y)}$$

Maximum Error:

(40) $$Maximum\;Error=max\left|y_{i}-{\overset{\wedge }{y}}_{i}\right|$$

**Figure 15:**
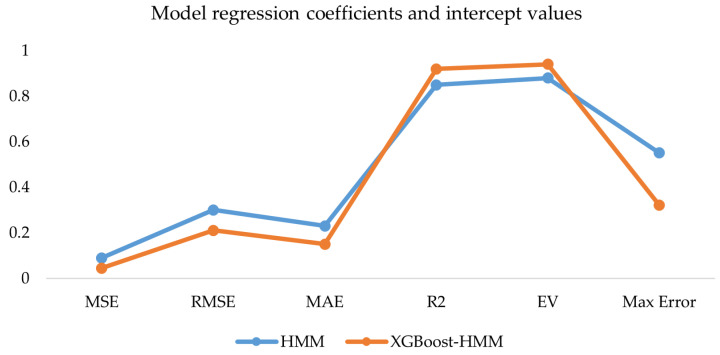
Model regression coefficients and intercept values.

The integrated driver data and vehicle data were used as inputs for the XGBoost-HMM in this study. The results were compared with the traditional HMM. The evaluation metrics are shown in Figure 15.

The results in Figure 13 show that both the HMM and XGBoost-HMM exhibit good performance in the road hypnosis identification regression task. XGBoost-HMM performs the best. The mean squared error and mean absolute error are the lowest among all models. The coefficient of determination (R^2^) and explained variance (EV) are the highest.

**Figure 16:**
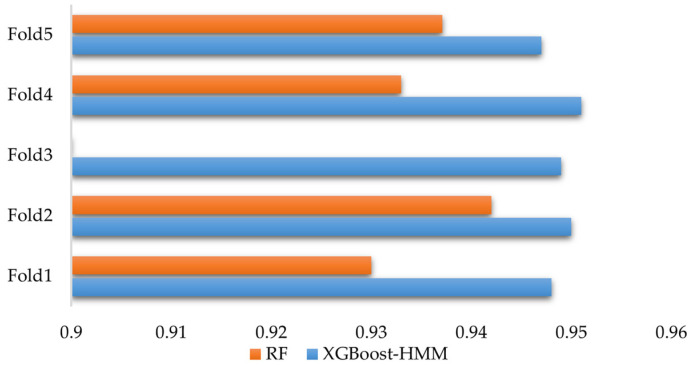
K-fold cross-validation accuracy.

In order to evaluate the performance differences of different classification algorithms when processing complex data. The random forest algorithm was introduced. The random forest algorithm is selected as an algorithm based on ensemble learning. Multiple decision trees of random forest are constructed and combined with their prediction results for classification. It has strong robustness and can effectively deal with high-dimensional data and noise. At the same time, it avoids overfitting. It also performs well when processing class-imbalanced data. It is relatively simple to adjust hyperparameters. It has a fast training speed. It is suitable for quickly verifying model performance. To further evaluate the model’s generalization ability, K-fold cross-validation (K-fold) was used to validate the model. The dataset used in this study includes driver data and vehicle data. It has high feature dimensions and complex data fusion. Five-fold cross-validation ensures adequate representation of each subset in both the validation and training sets with minimal computational cost. This allows for a more accurate evaluation of the model’s performance. The results are shown in Figure 16.

The key to the road hypnosis identification model lies in its ability to adapt to different data distributions. Five-fold cross-validation divides the data into five equal parts. One part is used as the validation set, and the remaining four parts are used as the training set. The average of the results from each validation is calculated. This evaluates the model’s stability and reduces the impact of random factors on the results. The experimental results show that the accuracy for each fold is very close. This indicates that the model’s performance fluctuates minimally on both the training and validation sets, demonstrating strong adaptability to different data splits and high stability.

The experimental results show that the accuracy of each fold of XGBoost-HMM is very close to and higher than that of random forest (RF) alone. This shows that the performance fluctuation of XGBoost-HMM on the training set and validation set is smaller than that of the RF model. This shows that XGBoost-HMM has strong adaptability to different data splits and high stability. The average value of all folds of XGBoost-HMM is the accuracy of the final model, which is 94.9%. It is higher than the accuracy of the RF model, which is 92.66%.

The calculation process is as follows:

(41) $$Accuracy=\sum_{i=1}^{K}Accuracy_{i}$$

In this case, $Accuracy_{i}$ represents the accuracy of the i−th fold, K represents the total number of folds.

XGBoost-HMM and random forest (RF) are compared in terms of computational efficiency. Specific indicators include training time, inference time (per sample), memory usage, and model size. We conducted experiments under the same hardware conditions (NVIDIA GeForce RTX4060, AMD Ryzen 7 7840 H with Radeon 780 M Graphics). The experimental results are shown in Table 3.

The training time of XGBoost-HMM is significantly lower than that of Random Forest. This shows that XGBoost-HMM has a clear advantage in training speed and can complete the training task faster. The inference time of XGBoost-HMM per sample is 0.0001 s. RF is 0.0006 s. This shows that XGBoost-HMM is more efficient in inference speed. Especially when processing a single sample, there is almost no delay. The memory usage of XGBoost-HMM is significantly lower than that of RF. Memory usage of XGBoost-HMM is significantly lower than RF. This indicates that XGBoost-HMM is more efficient in memory consumption. The model file of XGBoost-HMM is very small, significantly smaller than RF. This indicates that XGBoost-HMM is more compact in storage space. It can be more easily deployed on devices or systems with limited storage space and reduces the need for storage resources.

In summary, XGBoost-HMM shows better computational efficiency than random forest (RF) in terms of training time, inference time, memory usage, and model size.

The model is evaluated with multiple methods in this study. The importance of different modality features for road hypnosis identification in non-monotonous road environments is explained. The gain, coverage, and frequency metrics, as well as the SHAP and LIME explanation results, all show that EEG features such as beta and delta waves consistently rank highly in the importance evaluation. This indicates that EEG features are crucial for road hypnosis identification in non-monotonous road environments. These features significantly improve model performance and are applicable to a wide range of samples. Pupil diameter right and speed show strong performance across multiple importance metrics. This indicates that the fusion of eye movement and vehicle behavior data effectively enhances the model’s identification ability. The SHAP plot demonstrates the key roles of beta wave, delta wave, speed, and gamma wave in the model’s prediction. The LIME plot reveals the specific contributions of features such as speed, beta wave, and delta wave in individual samples. The model is evaluated with six metrics: mean squared error, coefficient of determination, root mean squared error, mean absolute error, explained variance, and maximum error. Additionally, the results from K-fold cross-validation show that the model’s accuracy is consistent across each fold. This indicates that the model exhibits strong stability and generalization ability under different data splits.

## 5. Conclusions

Specific driving experiment routes are selected for each participant with different fixed commuting routes in this study. Driver and vehicle data from 30 participants in road hypnosis states are collected through driving experiments. A road hypnosis state feature dataset is established. The driver data and vehicle data are preprocessed separately. Road hypnosis dynamic features are extracted from each dataset with power spectral density analysis, the sliding window method, and the point-by-point calculation method. The key features of the three types of data are fused into a unified feature vector through normalization and standardization methods. The HMM algorithm is optimized with the XGBoost algorithm. The fused feature vector is used as input to the XGBoost-HMM algorithm to construct the road hypnosis identification model. The model performance is measured with mean squared error, coefficient of determination, root mean squared error, mean absolute error, explained variance, and maximum error. The LIME-SHAP method is used to visualize and analyze the model’s decision-making process. The contribution of each feature to road hypnosis identification is revealed. The model’s performance is evaluated with K-fold cross-validation. The experimental results show that the XGBoost-HMM-based road hypnosis identification model for drivers developed in this study can effectively identify the road hypnosis state of drivers.

A new road hypnosis identification method is proposed in this study. Road hypnosis in any road scene is recognized. It can improve the accuracy of road hypnosis recognition in vehicles. It can improve the life recognition capabilities of active safety systems and smart cockpits in vehicles. It can also improve automobile safety. There are still some limitations in this study. A key limitation is the small sample size. This may affect the generalizability of the findings. The current study involved 30 participants. A larger sample size could be considered in future studies to improve the robustness and generalizability of the results. In addition, the experiments are only conducted under clear weather and good road conditions in this study. Different weather conditions (such as rain, snow, and smog), road conditions (such as slippery, bumpy roads), and time changes (such as night driving) can be simulated in the virtual driving environment. Vehicle and virtual driving experiments on road hypnosis under different weather and road conditions will be further considered in future studies.

## Figures and Tables

**Table 1:** The filter parameters.

Parameter	Low-Pass Filter	High-Pass Filter
$\mathrm{Sampling}\;\mathrm{Rate}\;(f_{s}$)	100 Hz	100 Hz
$\mathrm{Cutoff}\;\mathrm{Frequency}\;(f_{c}$)	8 Hz	0.1 Hz
Filter Order (M)	101	201
Window Function	Hamming Window	Hamming Window

**Table 2:** The model’s regression coefficients and intercept values.

Observation Sequence	Regression Coefficient	Value	Observation Sequence	Regression Coefficient	Value
Speed	$\beta_{1}$	0.231	Gamma	$\beta_{8}$	0.074
delta	$\beta_{4}$	0.078	Gaze Velocity	$\beta_{9}$	−0.007
theta	$\beta_{5}$	0.113	Pupil Diameter Left	$\beta_{10}$	0.083
alpha	$\beta_{6}$	0.004	Pupil Diameter Right	$\beta_{12}$	−0.179
beta	$\beta_{7}$	−0.112	IPD	$\beta_{14}$	0.071

**Table 3:** Comparison of computational efficiency between XGBoost-HMM and RF.

Index	XGBoost-HMM	RF
training time	0.0884 s	1.008 s
inference time (per sample)	0.0001 s	0.0004 s
memory usage	422.38 MB	537.8 MB
model size	0.11 MB	0.987 MB

## Data Availability

The data presented in this study are available on request from the corresponding author. The data are not publicly available due to privacy.
